# Preclinical Study on Novel Treatment Regimen for Elderly Patients with Drug-Sensitive Pulmonary Tuberculosis

**DOI:** 10.3390/ijms27219848

**Published:** 2026-11-03

**Authors:** Jie Zhou, Bo-Qian Wang, Yu-Jie Zhang, Jie Wang, Wen-Wen Sun, Xiao-Fen Liu, Xian-Ting Ding, Lin Fan

**Affiliations:** 1 Department of Tuberculosis, Shanghai Clinical Research Center for Tuberculosis, Shanghai Pulmonary Hospital, School of Medicine, Tongji University, Shanghai Key Lab of Tuberculosis, Shanghai 200433, China; 2 Institute for Personalized Medicine, School of Biomedical Engineering, Shanghai Jiao Tong University, Shanghai 200030, China; 3 Key Laboratory of Clinical Pharmacology of Antibiotics, National Health Commission of the People’s Republic of China, Institute of Antibiotics, National Clinical Research Centre for Aging and Medicine, Huashan Hospital, Fudan University, Shanghai 200040, China

**Keywords:** elderly patients, pulmonary tuberculosis, H_37_R_v_, AI-PRS, drug susceptibility test

## Abstract

Treatment for elderly patients with pulmonary tuberculosis (PTB) is challenging due to poor efficacy and tolerance. The aim of this study is to preliminarily screen candidate regimens for elderly patients with drug-sensitive PTB. Candidate regimens were initially screened using the artificial-intelligence-enabled phenotypic response surface (AI-PRS) platform and in vitro experiments. The hepatorenal cytotoxicity of drug combinations was assessed with HEK293T and THLE-2 cells. In the in vivo validation stage, aged mice with airway *Mycobacterium tuberculosis (Mtb)* infection received the novel regimens by oral gavage for 4 weeks, while isoniazid (H)/rifampicin (R)/ethambutol (E)/pyrazinamide (Z) served as the standard regimen control. Hepatorenal function was monitored during the treatment to assess drug safety, and pathological HE staining was also performed to evaluate liver and kidney damage. Bacterial loads in the lungs and spleens were evaluated using colony-forming unit (CFU) assays. After three rounds of AI-PRS analysis and MIC screening in vitro, three optimized treatment regimens were identified including delamanid (Dlm)/bedaquiline (Bdq)/pretomanid (Pa) combined with rifapentine (Rpt), pasiniazid (PAS-H) and levofloxacin (Lfx). After validation with an experiment on aged mice, the Bdq-containing regimen resulted in the mildest pulmonary inflammation and the lowest bacterial loads in lungs and spleens. The bacterial burden was similar between Bdq- and Dlm-containing regimens, which presented stronger bactericidal activity over the HREZ regimen. Regarding drug safety evaluation, the Bdq-containing regimen showed no marked hepatotoxicity and nephrotoxicity in our preclinical models, while other regimens showed variable abnormalities in serum biochemical indices. The Bdq/Lfx/PAS-H/Rpt regimen presented high efficacy and more favorable safety in aged mice compared with the standard regimen, considered as a promising preclinical candidate for elderly patients with drug-sensitive TB and deserving of further clinical trials for evaluation.

## 1. Introduction

Tuberculosis (TB) remains a challenge worldwide. Drug-sensitive tuberculosis has a treatment success rate of 88%, while that of rifampicin (RFP)-resistant TB is 71%, according to an updated report from the World Health Organization [1]. The six-month standard regimen for drug-sensitive TB is the recommended approach for most patients. However, in clinics, elderly patients often face poor efficacy of and tolerance to the six-month standard regimen. Elderly patients were reported to have adverse event incidence as high as 35.4%, which required treatment interruption and first-line drug discontinuation (with an occurrence rate of 26.2%), especially pyrazinamide (PZA) [2]. Additionally, elderly patients were reported to have a higher rate of anxiety, which is more likely to influence their tolerance of the entire course of TB chemotherapy [3]. Therefore, elderly TB patients have some distinct clinical features. Several studies have reported that elderly patients had higher mortality due to various reasons, such as diabetes, congestive heart failure, delayed diagnosis, low weight, and non-standard regimens [4,5]. One study reported that a high percentage (67.6%) of elderly patients were asymptomatic [6], while other cases had fever and inflammation-associated characteristics [7], which were likely to result in higher mortality or poor outcomes.

Given the clinical features and poor outcomes of elderly patients, there is currently a lack of treatment regimens specifically designed for elderly pulmonary TB patients. There are controversial views regarding the treatment of elderly patients. Some studies focused on treatment outcomes with the standard regimen (HREZ) in elderly patients. Some studies showed that the standard regimen was safe in elderly patients; however, higher mortality was observed in the standard regimen group, which may not be related to the regimen itself [8]. Another study showed that the PZA-containing standard regimen had more adverse events, allergic reactions, and antihistamine use [9]. Therefore, more physicians actually expect novel regimens specifically designed for elderly patients to be safer, better tolerated and less controversial.

Although many anti-TB drugs are currently available in clinics, screening the optimal regimen, especially for elderly patients, remains challenging due to the large number of drug–dose combinations. Traditional methods rely on fixed experimental designs, but the vast number of combinations, such as drug types and dosages, makes large-scale drug combination screening difficult. Since artificial intelligence (AI) has been widely applied in almost every aspect of medical research, it is possible to contribute to the design of suitable regimens for elderly patients. To overcome the limitations of traditional screening methods, researchers have developed the artificial-intelligence-enabled phenotypic response surface (AI-PRS) platform [10,11]. The platform is based on the characteristic of presenting a smooth parabolic surface in response to drug dose stimuli in cells or organisms, allowing researchers to complete mathematical modeling of the response surface by collecting only a small fraction of data from the total search space. Previous studies have confirmed that whether in macrophage models [12], preclinical in vivo experiments [13], or individualized treatment [14], PRS can efficiently guide the screening and optimization of anti-TB regimens. Wesley et al. [15] proposed BATCHIE, an orthogonal approach based on information theory and probabilistic modeling, which dynamically designs subsequent experimental batches based on previous results to rapidly identify highly effective and synergistic combinations. This method enables efficient, low-cost large-scale drug combination screening. In addition, Paul et al. [16] used a high-throughput drug combination screening platform called Combocat to screen 9045 combinations in neuroblastoma cells, providing a promising avenue for accelerating drug combination discovery. Although the aforementioned studies have achieved breakthroughs, research that directly employs AI for the screening and prediction of synergistic effects in anti-tuberculosis drug combinations is still considerably limited. Since AI has been widely applied in almost every aspect of medical research, it is possible to contribute to the design of suitable regimens for elderly patients.

**Figure 1:**
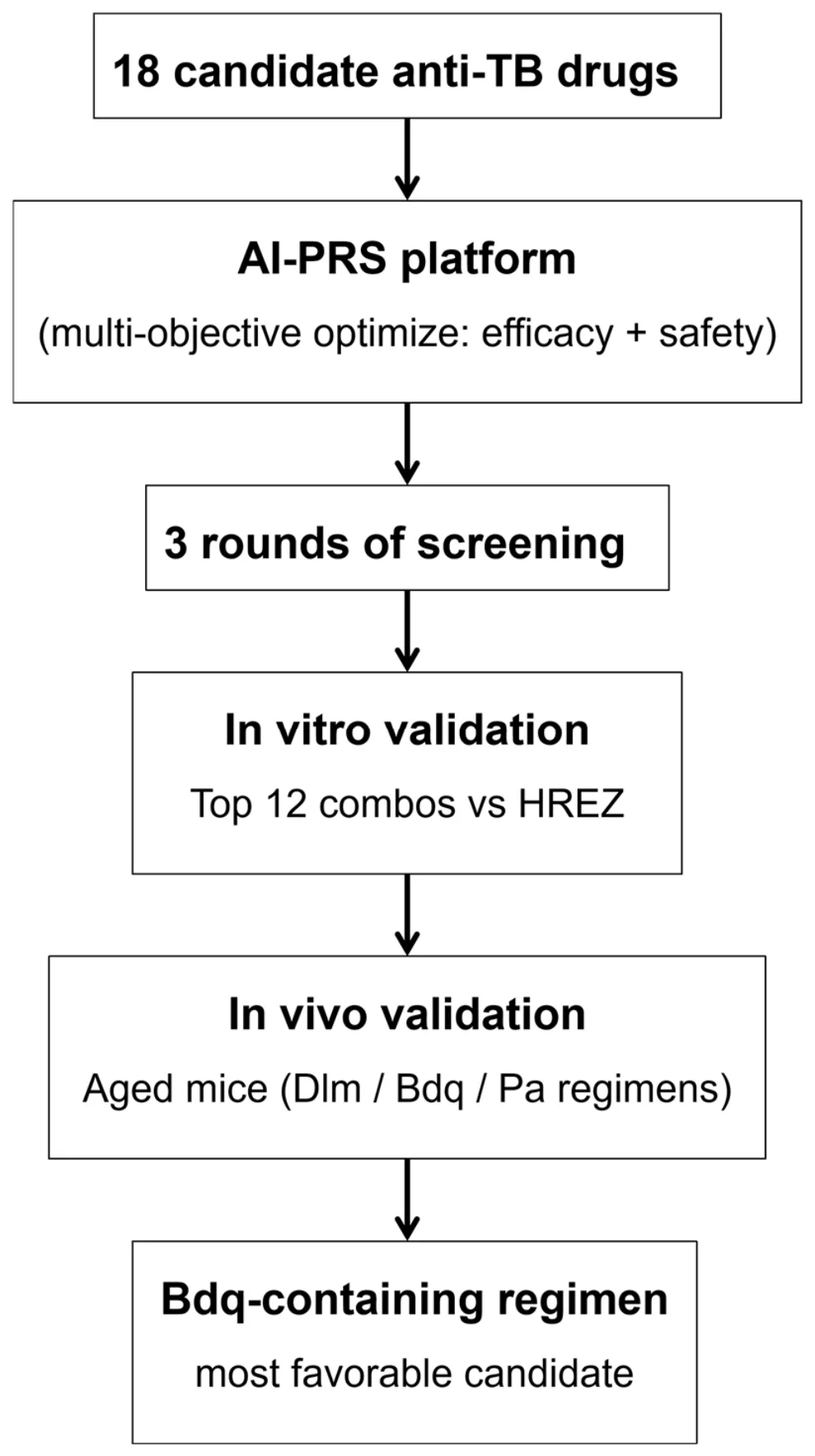
Schematic overview of the study design.

This study employed AI-PRS to design and screen optimal drug combinations for drug-sensitive PTB for potential use in elderly patients, aiming to identify the optimal regimen with superior efficacy and safety over the standard regimen (Figure 1). First, based on the previous screening scheme and clinical medication experience, the candidate combination regimens were screened after several rounds of in vitro experiments. Importantly, in addition to maximizing anti-*Mtb* activity, the in vitro optimization incorporated stringent hepatic and renal cytotoxicity constraints to account for the reduced organ functional reserve and increased susceptibility to drug-related toxicity that characterize elderly patients. Therefore, a multi-objective optimization function was newly proposed to achieve a more delicate balance between antibacterial efficacy and adverse cytotoxicity. Second, an elderly mouse model of *Mtb* airway infection was established. The optimal regimen with favorable efficacy and tolerance for elderly patients was screened and determined with the standard HREZ regimen.

## 2. Results

### 2.1. The AI-PRS Platform and First Round of Experiments

The AI-PRS platform integrates experimental design, high-throughput screening, computational modeling, and iterative optimization to efficiently identify drug combinations with superior efficacy-to-toxicity ratios from the vast combinatorial space. Starting from 18 candidate anti-TB drugs (Supplemental Table S1) applied widely in clinics, the platform progressively narrows the search space through three rounds of model-guided experimental screening. By incorporating drug–drug interaction terms and multi-objective optimization, the platform simultaneously optimizes for antimicrobial efficacy and host cell safety.

**Figure 2:**
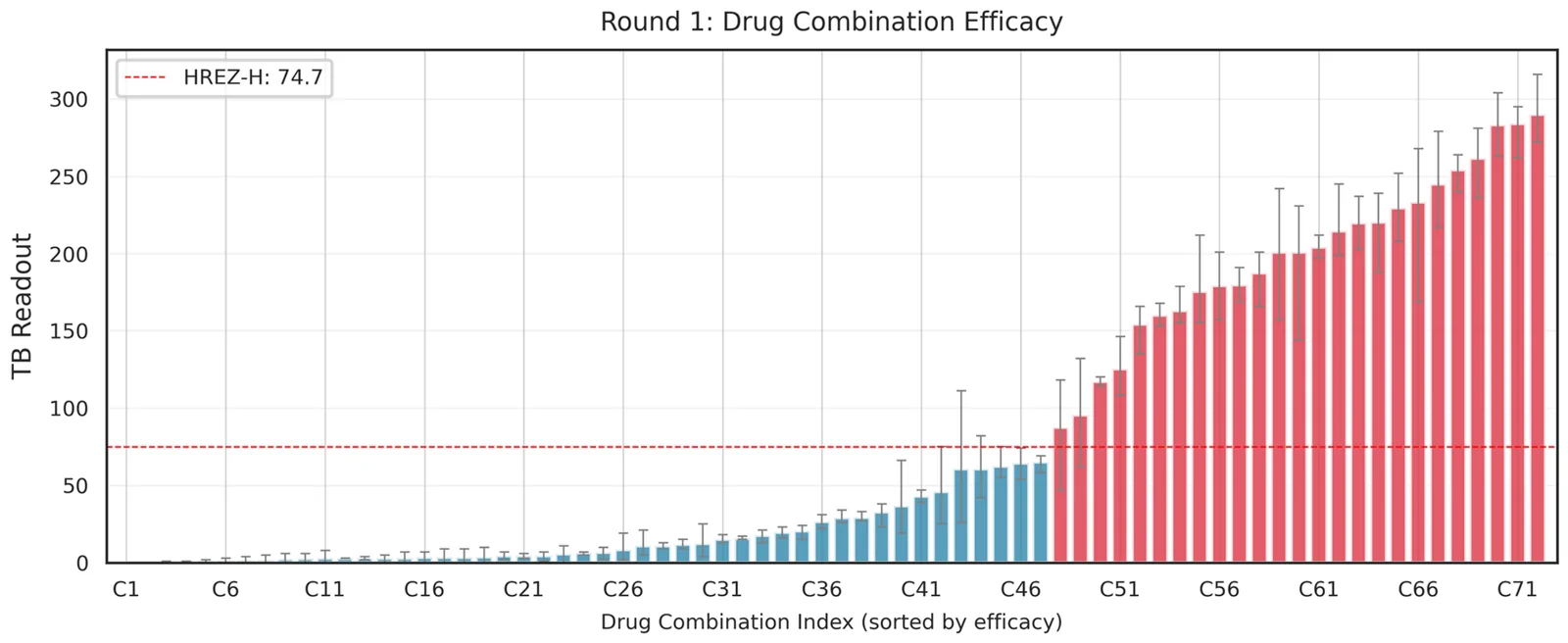
Round 1 Drug Combination Efficacy. TB readouts of the 72 four-drug combinations sorted by efficacy (*n* = 3, error bar indicating the min–max range of three replicates). Red dashed line: high concentration HREZ control. C1–C72 represent the 72 drug combinations ranked by efficacy. The blue and red bars indicate combinations with better and poorer efficacy, respectively, than the high-dose HREZ regimen.

The initial screening round evaluated 72 four-drug combinations across 18 anti-TB drugs. The experimental results demonstrated substantial variability in antimicrobial activity (Figure 2). This heterogeneity confirmed the importance of systematic screening to identify effective drug combinations.

**Figure 3:**
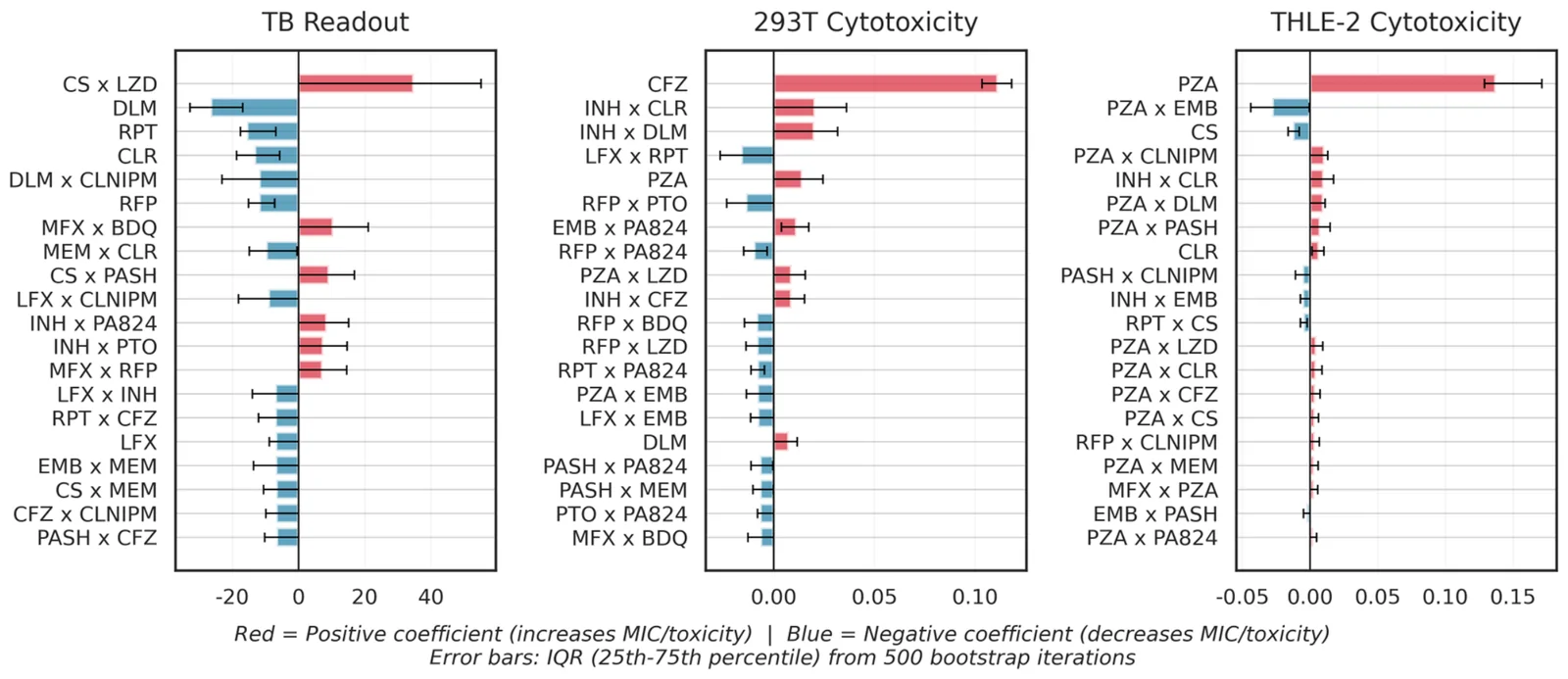
Bootstrap Lasso Coefficients. Top 20 features ranked by absolute coefficient magnitude for the TB readout model, HEK293T cytotoxicity model, and THLE-2 cytotoxicity model (C). Red bars indicate positive coefficients (increasing TB readout/toxicity), blue bars indicate negative coefficients (decreasing TB readout/toxicity). Error bars represent interquartile range (IQR, 25th–75th percentile) from 500 bootstrap iterations. Features include both main drug effects and pairwise interaction terms.

Bootstrap Lasso regression with interaction terms identified key features contributing to antimicrobial efficacy and cytotoxicity. The top twenty features ranked by absolute coefficient magnitude are presented in Figure 3. The main effects analysis revealed that DLM exhibited the strongest antimicrobial activity with a coefficient of −26.53 (IQR: −32.84 to −16.86), indicating that DLM was strongly associated with reduced TB readout. RPT ranked second with a coefficient of −15.62 (IQR: −17.55 to −6.81), followed by RFP at −11.83 (IQR: −15.11 to −7.20). Notably, several interaction terms (e.g., CS × LZD: 34.84) ranked among the most important features, indicating significant synergistic or antagonistic drug–drug interactions captured by the model.

For the HEK293T cytotoxicity model, the top features were primarily interaction terms, suggesting that nephrotoxicity is more strongly influenced by drug combinations than by individual agents alone. The THLE-2 hepatotoxicity model similarly identified interaction effects as the dominant contributors.

### 2.2. Iterative Optimization

**Figure 4:**
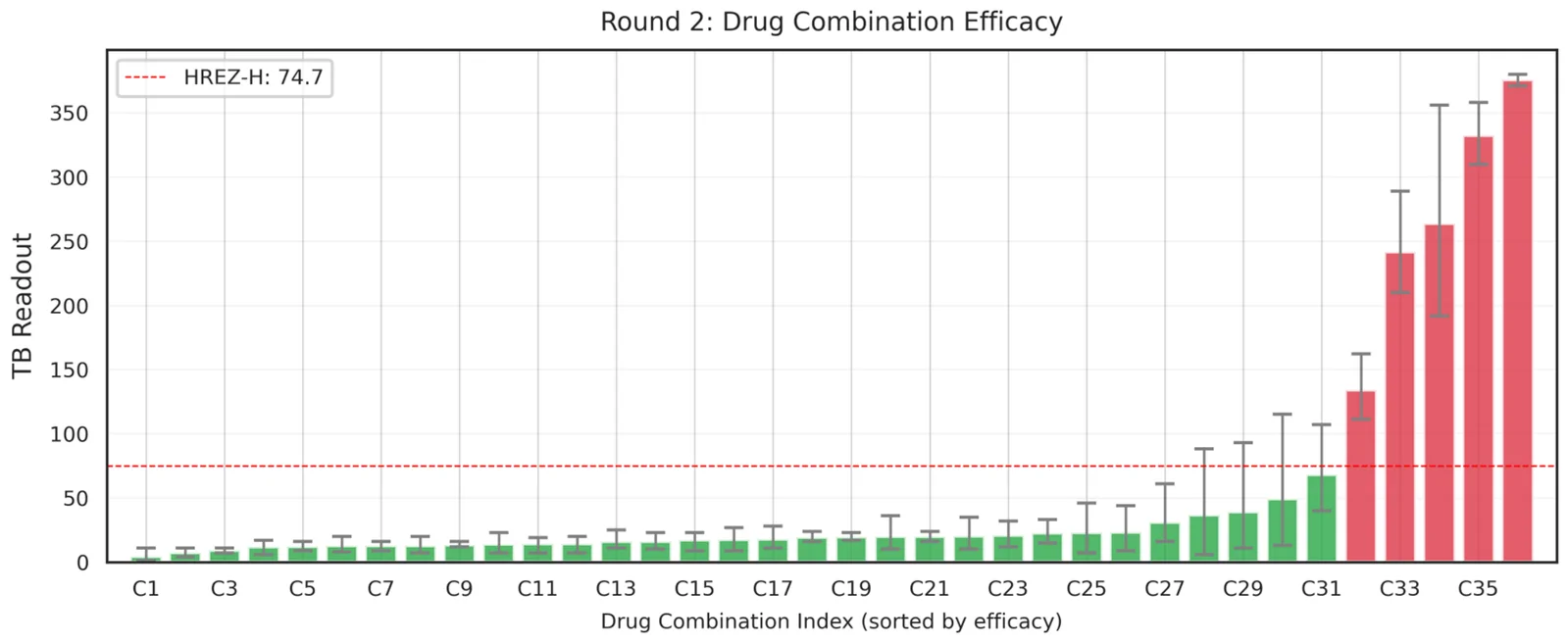
Round 2 Drug Combination Efficacy. TB readouts of the 36 drug combinations sorted by efficacy (n = 3, error bar indicating the min–max range of three replicates). Red dashed line: high concentration HREZ control. C1–C36 represent the 36 drug combinations ranked by efficacy. The green and red bars indicate combinations with better and poorer efficacy, respectively, than the high-dose HREZ regimen.

Based on the three models and the multi-objective optimization function, twelve drugs were selected for further evaluation in Round 2. Thirty-six combinations (eighteen three-drug and eighteen four-drug regimens) were experimentally validated in Round 2. As shown in Figure 4, these combinations demonstrated a marked improvement in antimicrobial efficacy compared to Round 1. The mean TB readout decreased to 55.1, representing a 31% reduction from Round 1. Several combinations achieved near-complete inhibition of *Mtb* growth, demonstrating the effectiveness of the model-guided selection strategy.

The HEK293T cytotoxicity remained well-controlled, with a mean response of 1.3% (IQR: 6.3%), indicating minimal nephrotoxicity across the tested combinations. The THLE-2 response averaged −6.3% (IQR: 5.9%), suggesting mild hepatocyte stimulation rather than inhibition in most combinations.

**Figure 5:**
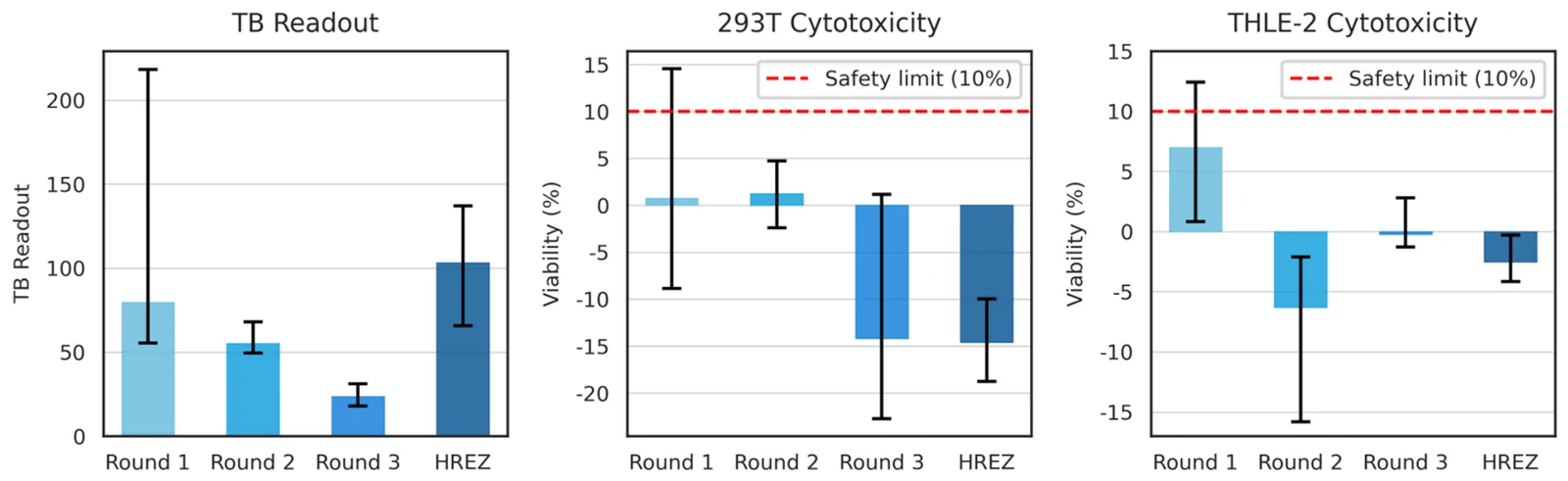
Optimization Trends Across Three Rounds. TB readout trend showing progressive 70% reduction (**left**). HEK293T renal cell nephrocyte response trend (**middle**). THLE-2 hepatocyte response trend (**right**). Error bars: IQR of combination means (*n* = 72, 36, 32, and 4 for Rounds 1–3 and HREZ, respectively). Red dashed lines: 10% safety threshold for cytotoxicity panels.

**Figure 6:**
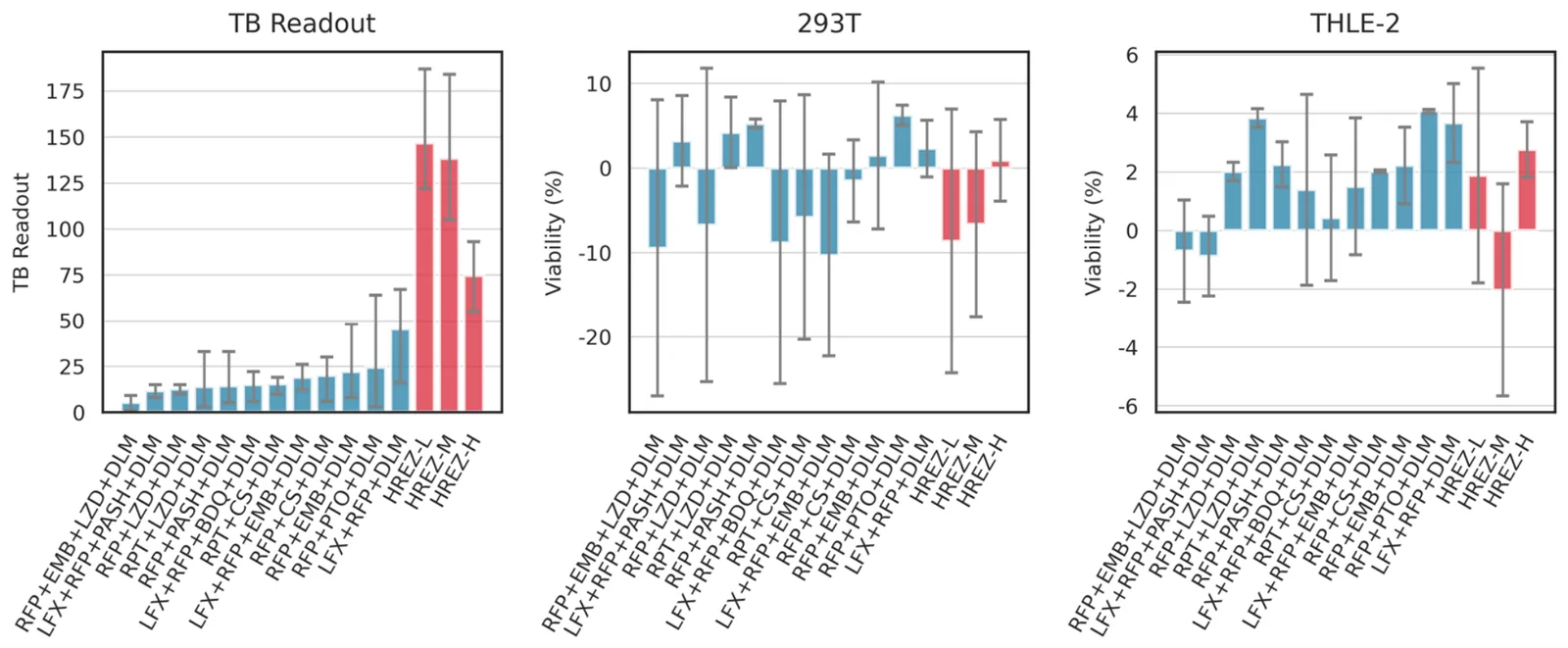
Round 3 Optimal Combinations versus HREZ. Comparison of the top 12 combinations and three HREZ concentration controls (L/M/H) across TB readout, HEK293T cytotoxicity, and THLE-2 cytotoxicity. Error bars: min–max range of triplicate measurements.

The Bootstrap Lasso models were then retrained, incorporating the complete experimental dataset from Rounds 1 and 2. Thirty-two novel combinations were generated for the next round of experiments. Average TB readout values decreased further to 23.7 (IQR: 17.0) in Round 3, representing a 70% overall reduction (Figure 5). In comparison, the HREZ standard regimen at three different concentrations exhibited an average TB readout of 103.1, confirming that the optimized combinations achieved significantly superior antimicrobial activity (Figure 6). Regarding safety profiles, HEK293T cytotoxicity showed moderate variation across rounds, with mean values of 0.8%, 1.3%, −14.2%, and −14.6% for Rounds 1–3 and HREZ, respectively. The THLE-2 hepatotoxicity remained low throughout the optimization process, with mean values of 7.0%, −6.3%, −0.2%, and −2.5%. All mean cytotoxicity values remained below the 10% safety threshold.

### 2.3. End of Optimization and Validation

With the addition of data from the third round, the Bootstrap Lasso models predicted no superior combinations to the tested ones, and, therefore, the optimization iteration ceased. We validated the top 12 combinations with the HREZ standard regimen at three concentration levels, referring to all four drugs at the low, medium, or high concentrations. In striking contrast, the optimized combinations demonstrated dramatically superior antimicrobial activity. As shown in Figure 6, the mean TB readout for the top 12 combinations was 18.3, substantially lower than the HREZ control groups. The leading candidate, Lfx/RFP/PAS-H/Dlm, achieved a TB readout of 12.1 [min–max: 8–15] with favorable cytotoxicity profiles. Other regimen candidates including Rpt/Cs/Dlm (15 [10-19]), RFP/Cs/Dlm (20 [6-30]), RFP/EMB/Dlm (22 [8-48]), and RFP/EMB/Lzd/Dlm (5 [1-9]) all demonstrated TB readout values substantially below the HREZ mean, with cytotoxicity within acceptable safety margins.

The AI-PRS platform successfully narrowed the search space from 18 drugs to 12 optimal 3-drug and 4-drug combinations, achieving superior efficacy while maintaining acceptable safety profiles. These regimens represent promising candidates for older pulmonary tuberculosis patients.

### 2.4. Therapeutic Efficacy and Safety Evaluation of Drug Regimens

**Figure 7:**
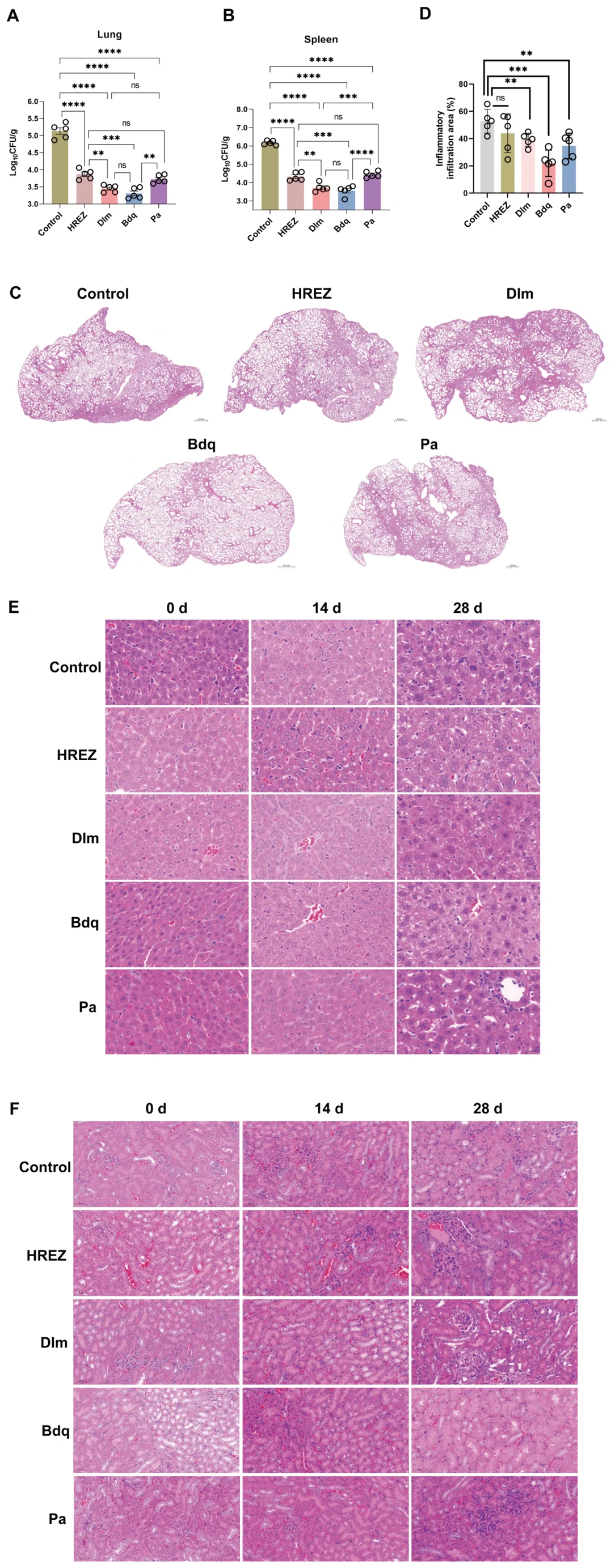
Therapeutic efficacy and safety evaluation of drug regimens. (**A**,**B**) Bacterial burden (CFU) in the lungs (**A**) and spleens (**B**) of 18-month-old C57BL/6 mice infected with *Mtb* via aerosol (~200 CFUs) and treated with different drug regimens by oral gavage for 4 weeks. (**C**) Representative H&E-stained lung sections showing histopathology of mice treated with different regimens. Scale bar = 625 µm. (**D**) Quantitation of inflammatory areas in the lungs of mice as in (**C**) (mean ± SD). (**E**,**F**) Representative H&E-stained liver (**E**) and kidney (**F**) sections from each group at days 0, 14, and 28 days of treatment, showing no obvious histopathological differences across groups. Scale bars = 25 µm (**E**) and 50 µm (**F**). *n* = 5. ** *p* < 0.01; *** *p* < 0.001; **** *p* < 0.0001, ns = not significant.

Following AI-PRS screening, the optimal regimens were Dlm/RFP or Rpt/PAS-H/Lfx. Research has shown that regimens using Rpt in place of RFP exhibit potent antimycobacterial activity, enabling a shorter treatment duration for patients with drug-susceptible PTB [17]. And, combined with our clinical observations, elderly patients tolerate Rpt better than RFP. Therefore, our novel treatment regimen used Rpt to replace RFP and employed Rpt/PAS-H/Lfx as the backbone, with Dlm incorporated as one of the candidate regimens for validation. Furthermore, Bdq and Pa are not inferior to Dlm in efficacy for TB treatment [18,19]. Thus, to evaluate the anti-TB efficacy and safety of different drug regimens in vivo, we designed regimens based on the Rpt/PAS-H/Lfx backbone and containing Dlm, Bdq, or Pa, respectively. We chose aged mice (18–20 months old), which were infected with H_37_R_v_ and subsequently administered the respective regimens by gavage. Based on the drug concentrations reported in previous studies, the administered concentration was adjusted to 80% of the conventional concentration in view of the physiological decline in aged mice. At the end of the observation period, various indicators were measured. CFU results showed that the *Mtb* burden in the lungs (Figure 7A) and spleens (Figure 7B) of mice in all treatment groups was lower than that in the control group. Among the regimens, the regimens containing Bdq or Dlm exhibited the greatest efficacy in reducing *Mtb* burden in the lungs and spleens of aged mice, with no significant difference between the two groups. This was followed by the HREZ regimen, whereas the Pa regimen showed relatively weaker effects. Compared with the control group, the Bdq regimen exhibited the mildest pulmonary inflammation in aged mice, characterized by markedly reduced immune cell infiltration and inflammatory lesions (Figure 7C,D). The Dlm- and Pa-containing regimens presented fewer inflammatory lesions than the HREZ and control groups, while no significant difference was found between the latter two groups. To evaluate the drug safety of each regimen, eight biochemical parameters, including alanine aminotransferase (ALT), aspartate aminotransferase (AST), and blood urea nitrogen (BUN), were measured in mouse serum to assess potential hepato- and nephrotoxicity. The results showed that the biochemical parameters in the Bdq regimen group were within the normal range, suggesting that the Bdq regimen group had the best drug safety without significant hepatorenal toxicity (Supplemental Figure S1A–H). However, the other groups (Dlm- and Pa-containing regimens and the control regimen) exhibited varying degrees of abnormalities in biochemical values.

HE staining revealed that the liver and kidney tissues of mice in all groups maintained structural integrity, without obvious morphological differences among groups (Figure 7E,F). The above results demonstrated that the Bdq-containing regimen exhibited optimal performance in reducing bacterial burden, alleviating pulmonary inflammation, and avoiding hepatorenal toxicity, positioning it as the most favorable candidate regimen for the treatment of PTB in elderly patients.

## 3. Discussion

Elderly TB patients are characterized by poor outcomes, with high treatment failure and mortality rates due to poor tolerance to standard regimens [20], and the sputum culture conversion rate was lower in PTB patients aged >65 years [21]. As elderly patients have decreased metabolic capacity in the liver, kidneys, heart and other organs [22], along with lower immune responses and stronger inflammation [23], it is essential to explore anti-TB regimens that are more appropriate for this population. However, to date, there remains a lack of anti-TB treatment regimens specifically designed for elderly patients. Because aging is frequently accompanied by reduced hepatic and renal functional reserve, drug regimens that are well tolerated in younger individuals may still pose substantial toxicity risks in older patients. Although the THLE-2 and HEK293T cell models used in this study do not directly reproduce cellular aging or the age-associated changes in drug disposition and organ function, cytotoxicity constraints in these models were incorporated into the AI-PRS optimization process to prioritize regimens with limited effects on host-cell viability. This strategy was motivated by the clinical need to identify treatment regimens with potentially improved tolerability in older patients, but the in vitro findings should be interpreted only as preliminary evidence of cytotoxicity under the experimental conditions tested. To further investigate the candidate regimens, we conducted in vivo validation in aged mice, including assessments of serum biochemical parameters and liver and kidney histopathology. Nevertheless, the aged mouse model also cannot fully reproduce the heterogeneity, comorbidities, polypharmacy, and age-related pharmacokinetic changes encountered in older patients. Therefore, the present findings provide preclinical evidence supporting further evaluation of the selected regimen, rather than direct evidence of its clinical safety in older patients.

The present study employed AI-PRS, and through three rounds of calculations, the optimal regimen was identified as Dlm/RFP or Rpt/PAS-H/Lfx. Dlm inhibits *Mtb* mycolic acid synthesis to exert bactericidal activity and is highly effective against first-line drug-resistant MDR-TB [24,25]. Rpt has potent activity against *Mtb* and is better tolerated than RFP in TB patients [26]. PAS-H exhibits high antibacterial activity [27], while Lfx not only shows strong bactericidal effects but also maintains efficacy against drug-resistant strains [28,29]. Although our in vitro experiment indicated that Dlm was superior to Bdq and Pa in bactericidal activity, this finding was consistent with a report using a Markov model simulation study [30]. However, in the clinical treatment of patients with MDR/RR-TB, Bdq-containing regimens presented higher rates of sputum culture conversion compared with Dlm [31] and even showed similar effectiveness to Bdq combined with Dlm [32]. Pa has a similar structure and anti-TB activity to Dlm [33]; thus, it remained unclear which drug-containing regimen was optimal for the treatment of elderly TB patients. Accordingly, based on the AI-PRS screening results, together with the drug characteristics described above and clinical observations, regimens containing Bdq, Dlm, or Pa on the Rpt/PAS-H/Lfx backbone were finally evaluated in aged mice for further screening.

Since PTB is primarily transmitted through the respiratory tract [34], we used aged mice infected with *Mtb* via the aerosol route to mimic natural infection and disease progression. The results indicated that the Bdq- and Dlm-containing regimens exhibited the greatest efficacy in reducing *Mtb* burden in both lungs and spleens, indicating strong treatment value, while the Pa-containing regimen presented lower anti-*Mtb* activity. All these three regimens presented obviously higher anti-*Mtb* effectiveness compared with the standard regimen (HREZ). Beyond antibacterial efficacy, the three regimens also differed markedly in pulmonary inflammation and hepatorenal toxicity. *Mtb* infection significantly promoted pulmonary inflammatory damage [35,36], whereas the Bdq-containing regimen showed less immune cell infiltration and inflammatory lesions, suggesting that it alleviates *Mtb*-induced immunopathological damage while exerting bactericidal effects (Figure 7C,D). Regarding hepatorenal function, the Bdq regimen exhibited no abnormalities in all eight serum parameters, whereas the other regimens showed varying fluctuations, though histopathological examination revealed no structural abnormalities in liver and kidney tissues across all groups, indicating that the Bdq regimen had a minimal impact on hepatorenal function. Collectively, the Bdq/Rpt/PAS-H/Lfx regimen was optimal in terms of achieving the best bactericidal activity, the mildest inflammation, and the lowest hepatorenal toxicity. This regimen showed superior efficacy compared with the standard-HREZ regimen, which will become a potential short-course regimen for drug-sensitive PTB in future clinical practice.

Although this study is the first to combine AI-PRS with both in vitro and in vivo experiments to preclinically explore novel anti-TB regimens intended for elderly patients with PTB, it still has four limitations. First, elderly patients are often accompanied by complicated factors such as polypharmacy and comorbidities, which cannot be fully recapitulated by the aged mouse infection model. Second, the standard treatment course for TB usually lasts more than 6–12 months, whereas the observation period in this experiment was relatively short. Third, nontuberculous mycobacteria (NTM) are intrinsically resistant to several first-line anti-TB drugs and may also contribute to poor outcomes in elderly TB patients [37,38]. As a potential confounding factor, NTM coinfection was not assessed in the aged mouse model in our study. Fourth, the sample size of the animal experiment was relatively small, and further validation with a larger sample size and more comprehensive safety evaluation is still warranted. Our future work should validate the conclusions in clinical elderly patients and extend the follow-up duration to further evaluate the long-term efficacy and safety.

In summary, the present study preclinically identifies a candidate optimal regimen for potential use in elderly patients with PTB through AI-PRS screening combined with validation in aged mice. The Bdq/Rpt/PAS-H/Lfx regimen showed the most favorable balance of efficacy and safety among the tested candidates in our preclinical models. These findings provide a novel candidate regimen with potentially improved efficacy compared with standard 6-month regimen, which deserves further validation by short-course regimen through clinical trials in elderly patients with PTB.

## 4. Materials and Methods

### 4.1. Cell Culture and Reagents

The human liver cell line THLE-2 (FH1249) was purchased from FuHeng Biology (Shanghai, China) and cultured in THLE-2 complete medium (FH-THLE-2). HEK293T cells (ATCC CRL-3216) were obtained from the American Type Culture Collection (ATCC, Manassas, VA, USA) and cultured in Dulbecco’s modified Eagle’s medium (DMEM, Cytiva, Logan, UT, USA) supplemented with 10% fetal bovine serum (FBS, Thermo Fisher Scientific, Waltham, MA, USA) and 100 U/mL penicillin–streptomycin. Both cell lines were cultured at 37 °C under a 5% CO_2_ atmosphere. Anti-TB drugs were obtained from two commercial sources. The following drugs were purchased from Aladdin (Shanghai Aladdin Biochemical Technology Co., Ltd., Shanghai, China): isoniazid (I104692), rifampicin (R105455), bedaquiline (B177578), levofloxacin (L157745), delamanid (D413211), pyrazinamide (P129219), ethambutol (E355168), and rifapentine (R304143). Isoniazid aminosalicylate (HY-B1048) and pretomanid (Pa, HY-10844) were purchased from MCE (MedChemExpress, Monmouth Junction, NJ, USA).

### 4.2. Minimum Inhibitory Concentration (MIC) Susceptibility Testing

Mycobacterium tuberculosis (*M. tuberculosis*, *Mtb*) H_37_R_v_ (ATCC#27294) was cultured at 37 °C to the logarithmic growth phase. The bacterial suspension was adjusted to a concentration of approximately 1 mg/mL and then diluted 1:50 with 7H9 liquid medium. Each well was inoculated with 100 µL of the diluted bacterial suspension. Drugs were dissolved and prepared at twice the desired final concentration, and 100 µL of the drug solution was added to each well, giving a final volume of 200 µL per well. The plates were incubated at 37 °C and examined at 7, 10, and 14 days post-inoculation. The presence of a visible white bacterial pellet at the bottom of a well was recorded as positive growth.

### 4.3. CCK-8 Cytotoxicity Assay

Cells were trypsinized, counted, and seeded into 96-well plates at a density of approximately 5 × 10^4^ cells per well in 100 µL of culture medium. The plates were incubated at 37 °C in a 5% CO_2_ incubator for 24 h. The supernatant was then removed, and 100 µL of various concentrations of the drug combinations was added to each well. Blank control wells (medium only) and negative control wells (cells without drug treatment) were also included. After 72 h of incubation, 10 µL of CCK-8 solution was added to each well, followed by incubation at 37 °C for 1 h. The absorbance was measured at 450 nm using a microplate reader.

### 4.4. Experimental Design for Drug Combinations

The initial drug library included eighteen anti-tuberculosis agents: levofloxacin (LFX), moxifloxacin (Mfx), isoniazid (INH, H), rifampicin (RFP, R), rifapentine (Rpt), pyrazinamide (PZA, Z), ethambutol (EMB, E), prothionamide (Pto), cycloserine (Cs), isoniazid aminosalicylate (also known as Pasiniazid, PAS-H), linezolid (Lzd), clofazimine (Cfz), bedaquiline (Bdq), delamanid (Dlm), imipenem/cilastatin (CLNIPM), meropenem (MEM), pretomanid (Pa), and clarithromycin (Clr).

The Uniform Design of Experiment principle was adopted in designing the initial round of experiments. Meanwhile, to prevent redundant exposure, each combination may include at most one drug from every single pair among RFP/Rpt, Dlm/Pa, and Lfx/Mfx. The first round of experiments comprised 72 four-drug combinations.

Each drug was set at three concentration levels. In preliminary experiments, no apparent cytotoxicity was observed at the drug concentrations used in the antibacterial assays. Therefore, the cytotoxicity assays were performed at 16-fold higher drug concentrations to further characterize cellular responses under higher in vitro exposure conditions.

### 4.5. The AI-PRS Platform

The AI-PRS platform proceeded iteratively: (1) initial design of experiments; (2) conducting the experiments to obtain efficacy data; (3) Bootstrap Lasso models were trained on accumulated experimental data to predict TB readouts and cytotoxicity for all possible combinations; (4) top-ranked combinations were selected based on the multi-objective optimization function for the subsequent round. The iteration ended when no more optimal combinations were predicted based on existing data and models.

A Bootstrap Lasso regression approach with interaction terms was employed to model the relationships between drug combinations and their biological responses. For each of the 500 bootstrap iterations, a random sample with replacement was drawn from the dataset, and Lasso regression with 10-fold cross-validation was performed. The optimal regularization parameter λ was selected based on the minimum mean squared error criterion. Model coefficients across all 500 bootstrap iterations were used to compute median effect estimates and interquartile range (IQR)-based confidence intervals.

The optimization objective was to identify drug combinations that simultaneously maximized antimicrobial efficacy and minimized adverse effects on hepatic and renal cell models. Given that elderly patients commonly have reduced hepatic and renal functional reserve and are, therefore, more susceptible to drug-related toxicity, stringent constraints on cytotoxicity in THLE-2 and HEK293T cells were incorporated into the optimization process to partially model the increased vulnerability to treatment-related organ toxicity observed in older patients. This was achieved by constructing a multi-objective optimization function:

Score = w_1_ × (TB readout) + w_2_ × |293T response| + w_3_ × |THLE-2 response|

where w_1_, w_2_, and w_3_ were set 0.5, 0.25, and 0.25, respectively.

### 4.6. Mice and Infection

Eighteen-month-old female C57BL/6J mice [39] were purchased from Shanghai Shengchang Bio-Tech Co., Ltd (Shanghai, China). Each mouse was infected with approximately 200 CFU of H_37_R_v_ via the aerosol route using a murine respiratory infection model. Then, a total of 25 mice were randomly assigned to 5 groups, with 5 mice per group. To evaluate the efficacy of the candidate TB treatment regimens, the mice were administered the drugs 3 weeks post-aerosol infection with H_37_R_v_. All drugs were dissolved in 0.5% carboxymethylcellulose sodium solution and then administered via oral gavage at a volume of 200 μL per time for 5 consecutive days per week. Based on the doses reported in previous studies [40,41,42,43,44,45,46,47,48,49], the drug doses used in this experiment are listed in Supplementary Table S2. After four weeks of treatment, the lungs and spleens of mice were collected for analysis of bacterial burden and histopathological changes. All animal experiments were approved by the Animal Care and Use Committee of Shanghai Pulmonary Hospital (approval number: K26-496).

### 4.7. Serum Biochemical Analysis

Blood samples (approximately 0.5–1 mL per mouse) were collected via cardiac puncture. The blood was allowed to stand at room temperature for 1 h, followed by centrifugation at 4 °C and 3000 g for 15 min. Serum was then separated and subjected to biochemical analysis. Liver function parameters, including alanine aminotransferase (ALT), aspartate aminotransferase (AST), total bilirubin (TBIL), albumin (ALB), and alkaline phosphatase (ALP), as well as kidney function parameters, including urea (UREA), creatinine (CREA), and uric acid (UA), were measured using an automated biochemical analyzer.

### 4.8. Hematoxylin-and-Eosin (H&E) Staining

Mouse lung, liver and kidney tissues were collected and fixed in 4% paraformaldehyde (PFA) at 4 °C for 24–48 h. Following fixation, the tissues were embedded in paraffin and sectioned. Staining was performed according to the manufacturer’s instructions for the hematoxylin-and-eosin staining kit (C0105S, Beyotime, Shanghai, China). Histopathological changes were observed under the microscope, and images were captured. The images were processed using KFSlideOS software (v1.1.2.0, KFBIO, Ningbo, China). Pulmonary histopathological scores were calculated as the ratio of the inflammatory infiltration area to the total lung area. Pathological changes in the liver and kidney were evaluated based on cell and tissue morphology.

### 4.9. Colony-Forming Unit (CFU) Assay

Mouse lungs and spleens were excised and washed with ice-cold sterile phosphate-buffered saline (PBS). The tissues were homogenized, and 100 µL of the homogenate was subjected to 10-fold serial dilutions (10^−1^ to 10^−3^) in sterile PBS. From each dilution, 100 µL was spread onto 7H10 plates, and each dilution was plated in triplicate. The plates were incubated at 37 °C for 3–4 weeks until colonies became visible, after which colonies were counted. The total bacterial burden per lung or spleen was calculated based on the dilution factor, and the results were expressed as Log_10_ CFU.

### 4.10. Statistical Analysis

Statistical analysis was performed using GraphPad Prism 9.0 (GraphPad Software, San Diego, CA, USA). Statistical significance between two groups was determined using a two-tailed Student’s *t*-test, while comparisons among multiple groups were performed using one-way analysis of variance (ANOVA). Differences were considered statistically significant at *p* < 0.05.

## Data Availability

The data that support the findings of this study are available from the corresponding author upon reasonable request.

## Supplementary Materials

The following supporting information can be downloaded at: https://www.mdpi.com/article/10.3390/ijms27219848/s1.
